# In Situ Dynamic Measurement of Blade Collision Warning Parameters for Coaxial Twin-Rotor Helicopters

**DOI:** 10.3390/s26092722

**Published:** 2026-04-28

**Authors:** Wenjie Zheng, Zurong Qiu

**Affiliations:** School of Precision Instrument and Opto-Electronics Engineering, Tianjin University, Tianjin 300072, China; tjbyzwj@163.com

**Keywords:** coaxial twin-rotor helicopter, blade collision early warning parameter, in situ dynamic measurement, multi-sensor fusion, millimeter-wave radar

## Abstract

In coaxial twin-rotor helicopters, the minimum blade tip distance may approach danger thresholds during rotor intersection under high-speed rotation and complex aerodynamic conditions, posing collision risks. This study proposes a multi-sensor fusion approach for measuring the blade collision warning parameter d, which maps the collision risk into a single evaluation metric and provides stable real-time outputs of phase, spatial position, and inter-blade distance under high-speed operational conditions. A collaborative measurement scheme integrating encoder-based phase detection, tip-tracking camera positioning, and millimeter-wave radar distance measurement was developed. A dynamic rotor motion simulation experimental platform with single-side rotation and rigid blades was constructed to validate the measurement performance under varying rotor speeds and blade tip distances. Experimental results indicate that measurement errors remain within ±1.87 mm, repeatability errors are below 0.67 mm, and the coefficient of variation is under 0.2%, confirming the accuracy and stability of the proposed method under dynamic conditions. Additional multi-speed experiments show that, over the tested rotational-speed range, the error of d remains within (−5.86 mm, 6.57 mm), although the fluctuation of the results increases moderately at higher speeds as the blade intersection duration becomes shorter. The proposed approach provides a laboratory-validated technical basis for blade collision risk assessment and future warning implementation in coaxial twin-rotor helicopters.

## 1. Introduction

Coaxial rotor helicopters offer unique advantages in low-speed heavy-lift operations, confined-space takeoffs and landings, and high-attitude maneuvers due to their compact layout, elimination of tail rotors, and high lift utilization efficiency [1,2,3]. However, under complex aerodynamic conditions and elastic deformation, the blade tip distance between the upper and lower rotors exhibits strong time-varying characteristics influenced by phase differences, blade swing, and pitch changes [4]. Under extreme conditions, the minimum blade tip distance may approach danger thresholds during rotor intersection, posing collision risks. To proactively mitigate this risk, it is necessary to establish an in situ real-time collision avoidance warning system. This system quantifies the danger margin at the instant of blade intersection in real time, using this metric as the criterion for flight control system intervention and adjustment to achieve closed-loop safety control. Within this warning framework, the key technical challenge lies in accurately obtaining the distance between the upper and lower rotor blade tips at the moment of intersection. Blade clearance serves as the core physical parameter characterizing collision risk, with its measurement accuracy and timeliness directly determining the reliability of the warning criteria. Traditional safety strategies primarily rely on structural redundancy design and ground-based offline inspections, lacking the capability for direct, real-time quantification of actual minimum blade tip clearance during helicopter flight operations. This limitation hinders the fulfillment of dynamic safety assessment requirements. Existing technologies for online blade clearance measurement typically employ single-sensor solutions, such as pure visual positioning or ultrasonic ranging [5]. However, these methods are susceptible to factors like occlusion, varying illumination conditions, ranging cycle limitations, and complex meteorological environments. They struggle to simultaneously meet the comprehensive requirements of real-time performance, high accuracy, and engineering deployment constraints in scenarios involving high-speed rotation and brief intersection periods. Further analysis reveals that under high-speed counter-rotating blade conditions, the measurement system must not only capture intersection events within millisecond time scales but also synchronously acquire blade phase information and spatial position parameters to eliminate measurement uncertainties caused by random phase differences. Therefore, to support online early warning during flight, an in situ real-time measurement method is required that can stably output three types of information—“phase, position, and distance”—under high-speed rotational conditions and uniformly map blade collision risks into a single measurement parameter representation.

The theory and methodology for in situ real-time measurement of blade collision early warning parameters not only provide scientific early warnings for dangerous approaches between upper and lower blades but also offer a critical basis for optimizing coaxial rotor structural design. The axial spacing between the upper and lower rotors [6] is a crucial parameter for coaxial rotor helicopters, and its optimization directly reflects the design level and performance refinement of the helicopter. Currently, the theoretical research on axial clearance design remains incomplete. Due to the inability to measure clearance at the moment of wingtip intersection during operation and the lack of operational experimental data, design relies largely on empirical formulas, incorporating sufficient redundant clearance to prevent blade collisions. This paper proposes an optimization objective for axial pitch: the minimum tip clearance during intersection to prevent collision and the maximum tip clearance during intersection to ensure reasonable clearance for the coaxial rotor structure. Achieving this objective requires not only theoretical analysis of blade deformation patterns under various complex operating conditions and improved structural stiffness, but most critically, the capability for real-time field monitoring of the distance between upper and lower blade tips during different flight conditions. This enables the acquisition of blade collision warning parameters. Therefore, real-time field measurement of wingtip distance is the key technological assurance for optimizing axial spacing, advancing helicopter design standards, and obtaining blade collision warning parameters.

In addition to direct blade tip distance measurement, non-contact optical techniques and low-intrusiveness onboard sensing have also been investigated for rotor blade structural characterization and dynamic state monitoring. Bernardini et al. [7] employed photogrammetry for the experimental identification of structural and inertial properties of helicopter rotor blades, demonstrating the value of non-contact optical reconstruction for rotor-blade measurement. Serafini et al. [8] further developed a non-invasive dynamic measurement system for helicopter blades and evaluated its in-flight performance, showing the feasibility of low-intrusiveness onboard monitoring under operating conditions. These studies provide important support for rotor blade non-contact measurement and structural health monitoring, but they mainly focus on blade structural characterization and dynamic response monitoring rather than instantaneous blade tip distance estimation at rotor intersection. Recent studies also showed that reliable dynamic parameter identification under sweep-frequency and nonstationary excitation is important for structural response interpretation in aerospace applications [9]. In parallel, microwave-based sensing has been actively studied in high-speed rotating machinery for blade tip timing and tip distance measurement [10,11], owing to its non-contact nature, short response time, and adaptability to harsh environments. These developments suggest that microwave/radar sensing has clear potential for rotor blade tip distance measurement, although its application to coaxial rotor intersection scenarios still requires phase-locked triggering and coordinated use with position-related information.

Current methods for measuring blade tip distance are primarily categorized into indirect and direct measurement techniques. Typical indirect measurement methods include strain measurement [12] and acceleration measurement. Strain measurement calculates the blade’s strain deflection curve by fitting and decoupling the strain values measured at different positions on the blade [13], providing a basis for blade structural evaluation and performance improvement. Computers perform strain continuity using demodulated finite-point measurements to derive strain variation equations, thereby calculating tip displacement to obtain blade tip distance. The acceleration measurement method involves installing an accelerometer at the blade tip. By measuring acceleration data at the tip and transmitting the signal via a slip ring to the airframe, the displacement value is obtained through secondary integration by the signal analysis and processing system. Direct measurement methods utilize distance sensors (cameras [14,15], ultrasonic, electromagnetic waves, etc.) to directly acquire the blade tip distance. André Bauknecht proposed a blade tip displacement measurement method based on onboard monocular vision [16]. By combining high-speed imaging with optical marker recognition, it enables non-contact acquisition of blade tip motion during flight, addressing the limitations of traditional ground-based measurement methods in real-time rotor dynamics characterization. However, optical imaging is susceptible to the strong occlusion characteristics of twin rotors, often requires carefully arranged installation positions, and suffers from poor ranging stability. Ultrasonic sensor-based tip distance measurement is under investigation. Yaohuan Lu achieved non-contact measurement of inter-blade spacing during rotation by transmitting and receiving ultrasonic signals between the upper and lower rotor blades [17]. Introducing coded excitation and correlation processing algorithms enhanced the ranging robustness under high-speed rotation, mitigating signal interference and measurement ambiguity in coaxial rotor configurations. However, ultrasonic propagation speed in atmospheric conditions is relatively slow compared to blade tip velocities, making it difficult to support real-time measurement during rotor intersection. Furthermore, external factors such as rain, snow, fog, dust, and gas disturbances between dual rotors significantly degrade ultrasonic ranging accuracy in practical environments, posing challenges for engineering applications.

The above analysis indicates that no single existing sensing method can independently provide all the information required for in situ real-time evaluation of blade collision risk under coaxial rotor intersection conditions. In particular, the key difficulty is not only distance acquisition itself but also the synchronized determination of blade phase, spatial position, and instantaneous blade tip distance within a very short intersection window. Therefore, this paper presents a multi-sensor fusion scheme based on angle encoder, tracking camera, and millimeter-wave radar. The concept of blade collision early warning parameter is used as a single parameter evaluation method. The measurement method design, measurement system construction, and measurement error analysis are carried out around the blade collision warning parameter.

## 2. A Definition of the Blade Collision Early Warning Parameter

### 2.1. Geometric and Kinematic Characteristics of Coaxial Rotor Systems

**Figure 1:**
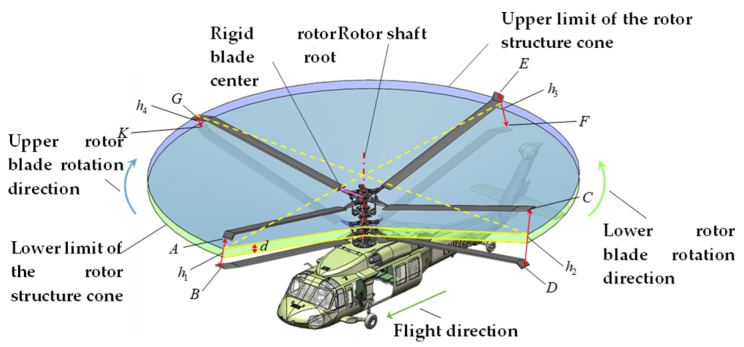
Conical trajectories of the rotor blades for coaxial rotor helicopters.

Taking an eight-bladed coaxial rotor helicopter as an example, as shown in Figure 1, within the 360° rotation range of the rotor blades, there are eight intersections of the blades with a phase difference of 45°. Taking four intersection points at a given moment as an example, points A, C, E, and G in the figure represent observation points at the tip of the upper rotor blade, while points B, D, F, and K represent observation points at the tips of the lower rotor blades. The blade tip distance of a coaxial rotor system refers to the real-time distance between the observation points at the tips of the upper and lower rotor blades when they intersect, denoted as $h_{1}$, $h_{2}$, $h_{3}$, and $h_{4}$ respectively. Since the blades are not perfectly rigid materials, they undergo deformation during rotational flight. The real-time blade tip distance between the upper and lower blades, which rotate in opposite directions, varies randomly at different phase-intersection moments due to factors such as flight speed, flight direction, and meteorological conditions. In the helicopter shown in Figure 1, the upper rotor rotates clockwise while the lower rotor rotates counterclockwise. The nose points in the forward direction, and wind speed and other meteorological effects are neglected. When the blades intersect at the phase shown in Figure 1, with flight speed $\vec{v}\neq 0$, the relative airspeed is maximum at point C on the upper rotor and minimum at point G, while on the lower rotor, point K has the maximum airspeed relative to the air, and point D has the minimum. At this moment, the lift exerted by the air on the rotor blades at points G and D is minimal, resulting in minimal deformation, whereas the lift exerted by the air on the rotor blades at points C and K is maximal, resulting in maximal deformation. Therefore, under the above ideal conditions, tip distances $h_{2}>h_{4}$ and $h_{1}>h_{3}$ occur. In practice, however, varying combinations of meteorological conditions and flight parameters induce complex rotor deformations such as flutter, oscillation, and torsion. Consequently, at the intersection points of coaxial twin rotors operating at different phases, the real-time blade tip distance h not only fluctuates randomly in magnitude but also exhibits random variations in the position of its projection along the coaxial axis. Consequently, for reliable collision risk assessment as a basis for safety warnings, both the magnitude of the tip distance and its position relative to the coaxial axis must be considered.

### 2.2. A Definition of the Collision Early Warning Parameters

To assess the likelihood of collisions occurring between the upper and lower rotor blades, this paper proposes the establishment of upper and lower limits of the rotor structure cone to delineate the danger zone. Specifically, using the coaxial axis as the reference, when the upper and lower rotors intersect, the lowest point of each upper rotor blade tip (point G at the moment shown in Figure 1) is connected to the center point of the non-rigid rotor blade swing hinge or the center point of the rigid rotor blade root (hereafter referred to as the blade root center point) (point M in Figure 1) as the generatrix. A cone surface coaxial with the common axis is constructed, termed the upper limit of the rotor structure cone. When the upper and lower rotor blades intersect, the cone surface passing through the highest point of each lower rotor blade tip (point K shown in Figure 1) and parallel to the upper limit of the rotor structure cone is termed the lower limit of the rotor structure cone. The distance d between the upper and lower limits of the rotor structure cone represents the collision avoidance margin. A larger d value indicates a lower probability of blade collision, while a smaller d value indicates a higher probability. This distance d is defined as the blade collision warning parameter. This parameter serves as the target variable throughout subsequent measurement and experimental verification processes.

For engineering applications, the threshold for blade collision warning parameters should not be set as an empirical constant independent of the rotor configuration and operating conditions but should instead be determined jointly by the physical collision boundary and the minimum safety margin under critical operating conditions. Specifically, this paper considers d=0 as the physical danger boundary, i.e., the limit state where the conical surfaces of the upper and lower rotor structures are exactly tangent and the blade tip distance is zero; however, to meet the requirements for real-time warning and flight control intervention, the actual warning threshold should be set on the safe side of this physical boundary. Therefore, the engineering warning threshold $d_{th}$ can be determined based on the minimum parameter value obtained from a set of typical critical operating conditions that still maintain contact-free operation and further incorporate safety margins required for measurement uncertainty, installation deviations, dynamic disturbances, and control response time; that is, when $d\leq d_{th}$, the system is deemed to have entered a warning state, where

(1) $$d_{th}=d_{\min,\Omega }^{safe}+\Delta_{meas}+\Delta_{inst}+\Delta_{dyn}+\Delta_{ctrl}.$$

Here, $d_{\min,\Omega }^{safe}$ represents the minimum collision early warning parameter measured or calculated under verified critical operating conditions. $\Delta_{meas}$, $\Delta_{inst}$, $\Delta_{dyn}$, and $\Delta_{ctrl}$ represent the safety margins corresponding to measurement error, installation error, dynamic fluctuations, and the time reserve for flight control intervention, respectively. This setting method ensures that the threshold has a clear physical meaning while also reflecting condition-dependent characteristics, thereby better meeting the requirements of engineering warning applications. Existing research on coaxial rotor systems indicates that the minimum tip clearance is a key metric for assessing safety, with unfavorable values typically occurring during critical flight conditions such as low-speed flight and descent, as well as under adverse load redistribution conditions.

Equation (1) also indicates that the required measurement accuracy should be evaluated relative to the overall safety margin included in $d_{th}$. From the perspective of the measurement system, the uncertainty in the measurement of early warning parameters should be significantly lower than this safety margin. By considering both appropriate uncertainty and response time margins when determining actual thresholds for specific rotor configurations and operating conditions, the proposed method provides a sound measurement basis for threshold discrimination.

### 2.3. Measurement Constraints and Technical Requirements

This paper investigates the blade collision warning parameter d (rotor speed: 420 r/min; measurement range: 99–990 mm; measurement error requirement: less than 15 mm), focusing on the following research areas:

Research on in situ real-time measurement methods for blade collision warning parameters. The measurement of blade collision warning parameters involves identifying the moment of upper and lower blade intersection during helicopter flight to trigger the measurement system. It enables real-time measurement of blade tip distance and the spatial position of either the upper or lower blade tip. Based on these measurements, the upper and lower limits of the rotor structure cone are constructed to calculate the blade collision warning parameters. The blade intersection timing is determined by installing angle sensors at appropriate positions on the dual-rotor transmission mechanism to measure the phase information. The phase positions of each blade on the upper and lower rotors are then calculated based on the transmission system’s gearing relationship, yielding the intersection timing. Real-time blade tip distance measurement employs millimeter-wave radar-based electromagnetic ranging, with sensor modules mounted at tip observation points (points B, D, F, and K in Figure 1). Real-time tip position measurement employs visual positioning. A visual measurement module is mounted on the hub, incorporating tracking targets and positioning algorithms to achieve real-time tip tracking. This converts high-dynamic measurements into relative stationary measurements, establishing a unified coordinate reference for the system. The data processing terminal integrates measurement results from all modules to calculate and output collision warning parameters for each blade intersection.

## 3. Overall Design and Components of the Measurement System

### 3.1. System Architecture Overview

**Figure 2:**
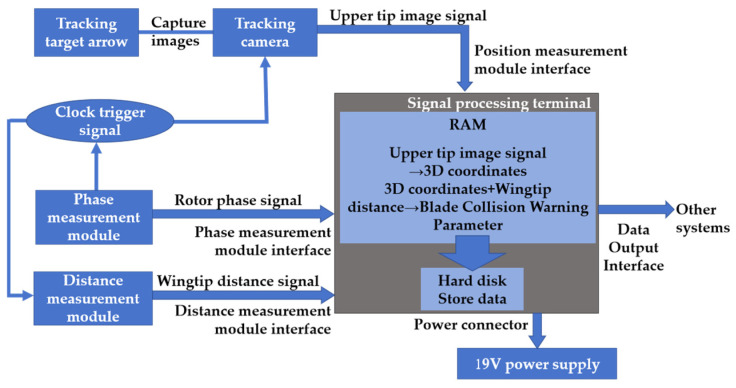
Measurement system architecture and phase-triggered data-fusion workflow.

The blade collision warning parameter measurement system primarily comprises sensor modules: phase measurement, distance measurement, and position measurement modules, along with a signal processing terminal. Phase measurement utilizes an angular encoder, distance measurement employs millimeter-wave radar, and the position measurement module includes a tracking camera and a tracking target. As shown in Figure 2, the system diagram summarizes not only the hardware configuration and signal transmission relationships but also the main data-fusion and processing workflow of the proposed method. First, the phase measurement module outputs the rotor phase signal, from which the signal processing terminal determines the theoretical blade intersection instant and generates the corresponding clock trigger signal. Under this phase-triggered mode, the distance measurement module and the position measurement module are synchronously activated within the same blade intersection event. The distance measurement module acquires radar echo data and outputs the blade tip distance information, while the position measurement module captures the image of the tracking target vector at the rotor tip and transmits it to the signal processing terminal. In the camera branch, the terminal performs image-based tip localization and converts the tracking target image into the three-dimensional coordinates of the upper blade tip. These coordinates are then mapped into the unified measurement reference frame for subsequent fusion with the ranging results. In the radar branch, when multiple distance samples are obtained within one intersection event, the sample whose phase is closest to the theoretical blade intersection phase is selected as the effective tip distance measurement result. Finally, the synchronized phase, position, and distance data are fused in the signal processing terminal to reconstruct the corresponding blade intersection state and calculate the blade collision warning parameter d. The calculated parameter is then stored on the hard disk of the signal processing terminal for subsequent analysis and retrieval.

### 3.2. Encoder Measurement Module

**Figure 3:**
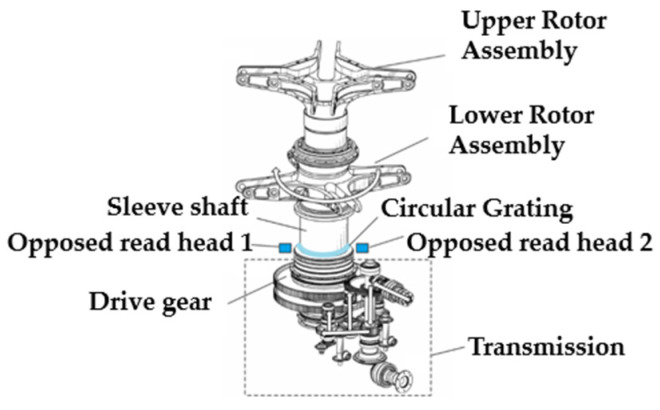
Coaxial rotor transmission system and angle sensor layout.

Blade phase identification is the critical factor in determining the moment of blade intersection for coaxial rotor helicopters, and it ensures that measurements of blade tip distance and tip position are performed at the correct phase moment. Since the upper and lower rotors of a coaxial rotor helicopter rotate in opposite directions at the same speed, blade intersection between them occurs only at specific phases. This paper proposes an angle-sensing-based phase identification method, as illustrated in Figure 3. The engine drives the dual rotors to rotate in opposite directions through a transmission mechanism. Since the transmission ratio of this system is fixed, the phase relationship between the upper and lower rotors relative to the transmission system remains constant [18]. Consequently, the phase at which the blades of the dual rotors intersect relative to the helicopter fuselage is fixed. Therefore, by determining the phase zero point relative to the fuselage, the phase at each intersection point can be obtained. When the rotational phase at any point along the transmission chain from the engine to the rotor is known, the rotational phase of the coaxial rotor blades can be derived based on the transmission ratio relationship between that point and the rotor. Therefore, the solution involves installing a circular encoder angle sensor system on the sleeve shaft and mounting a diameter reading head on the fuselage to collect data, thereby obtaining the rotational phases of the upper and lower rotors as well as the timing of each blade’s intersection.

The phase measurement module employs an absolute circular encoder with an absolute zero position. When the measured shaft system rotates, the near-instantaneous image of the encoder scale is captured by the read head. Through a digital signal processor combined with a correction algorithm, this image yields a high-resolution, precise position, ensuring the read head’s measurement accuracy.

### 3.3. Tracking Camera Measurement Module

**Figure 4:**
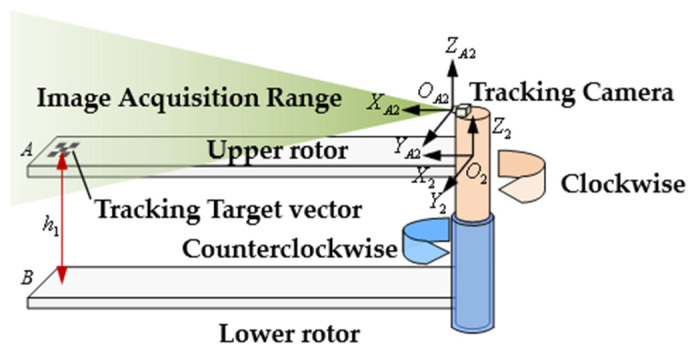
Visual positioning-based tip position measurement solution.

To define the upper and lower limits of the rotor structure cone, spatial positioning of the upper blade tip observation points is required to define a unified reference frame. This paper proposes a vision-based blade tip position measurement method to determine the location of the upper blade tip observation points, as shown in Figure 4. The tracking camera is fixed to the upper rotor hub, and rotates synchronously with it, maintaining a rigid relative position. The camera’s field of view is directed toward the corresponding blade tip, ensuring it remains within the camera’s image capture range during flapping, twisting, and pitch-angle movements. This enables measurement of the blade tip position at each blade intersection.

Spatial positioning can be achieved using monocular or stereo vision. While stereo vision offers higher positioning accuracy, its algorithms are more complex, it requires larger dimensions and weight, and it operates at slower measurement speeds. Therefore, this paper employs a monocular vision approach combined with a tip-tracking target marker to locate the blade tip. This method simplifies the algorithm, reduces size and weight, and enables higher measurement speeds, making it better suited for the blade tip positioning task. The tracking camera captures images of the tracking target vector at the moment of blade intersection. Image processing then outputs the observation point coordinates, completing the tip position measurement and providing a unified coordinate reference for the entire measurement system.

The tracking camera follows a standard monocular imaging model with lens-distortion correction, and its intrinsic and distortion parameters are obtained using Zhang’s calibration method [19]. In this study, the calibration output is used to establish the mapping from image measurements to the three-dimensional tip-position coordinates required for the subsequent fusion process, rather than to develop a new camera model.

**Figure 5:**
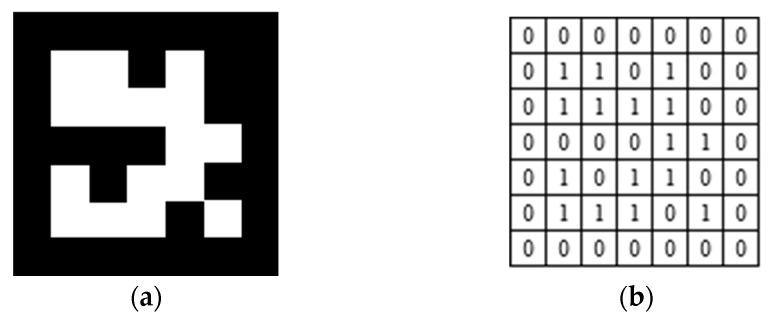
ArUco marker used for blade tip tracking. (**a**): marker appearance; (**b**): binary encoding structure.

The blade tip-tracking target vector adopts ArUco markers [20], as shown in Figure 5. ArUco markers provide clear border corners and internal binary codes, which make them suitable for robust target identification and pose estimation in the present measurement system.

In the present implementation, the ArUco target is detected in the image captured at the blade intersection instant, and the corner correspondences are combined with the calibrated camera parameters to estimate the target pose through a standard PnP procedure [21,22]. The obtained pose is then converted into the three-dimensional coordinates of the upper blade tip observation point in the camera coordinate system. These coordinates are subsequently used in the unified coordinate mapping and warning-parameter reconstruction described in Section 4 and Section 5.

To quantitatively evaluate the calibration quality and positioning accuracy of the tracking camera positioning module, this paper validates the positioning module on a Hexagon Global Classic SR coordinate measuring machine (CMM) platform after determining the positioning scheme. The camera model used is the iRAYPLE-A7900MG13 (Huarui Technology/iRAYPLE, Hangzhou, China), which employs a CMOS sensor with a resolution of 4096 × 2160, a pixel bit depth of 12 bits, a pixel size of 3.45 μm × 3.45 μm, and an exposure time range from 48 μs to 1 s. The lens model is MH1228X, with a focal length of 12 mm and a field of view of 70.5° × 59.8° × 46.3°. This paper employs Zhang’s checkerboard calibration method to estimate intrinsic and distortion parameters by capturing checkerboard images under various poses and evaluates the accuracy using a standard experimental procedure adopted in existing studies of optical measurement systems [21]. The calibrated camera intrinsic parameters are as follows: $f_{x}=3597.9790$, $f_{y}=3598.7629$, $c_{x}=2029.1367$, and $c_{y}=1075.7546$. The main distortion coefficients are $k_{1}=-0.07830$, $k_{2}=0.14253$, and $p_{1}=p_{2}=0$. After calibration, the mean reprojection error is below 0.1 pixels, indicating that the estimated intrinsic and distortion parameters are sufficiently accurate for subsequent blade tip positioning.

Based on the calibrated camera model, the tracking target is driven to perform simulated blade tip motion on the CMM platform, and the positioning errors of the module are evaluated along the flapping, flutter, and torsional directions. The corresponding error ranges are (−0.60, 0.50) mm, (−0.01, 0.01) mm, and (−0.79, 0.73) mm, respectively. Since the blade collision early warning parameter investigated in this study mainly depends on the blade tip coordinate in the flapping direction, the effective positioning error of the position measurement module adopted in the subsequent analysis is taken as (−0.60, 0.50) mm.

### 3.4. Millimeter-Wave Radar Measurement Module

The blade tip distance measurement module is used to obtain the spatial distance between the upper and lower blade tip observation points in situ and in real time during high-speed blade rotation. Millimeter-wave radar is a type of active electromagnetic ranging sensor operating in the millimeter-wave frequency band, typically utilizing carrier frequencies between 30 and 300 GHz. It achieves non-contact distance measurement by transmitting modulated electromagnetic waves and receiving target echo signals. Among these, the Frequency-Modulated Continuous Wave (FMCW) scheme has become the most widely adopted modulation method in millimeter-wave ranging radars due to its high ranging accuracy, continuous measurement capability, and high system maturity [22]. This scheme achieves target distance inversion by linearly modulating the transmitted signal and utilizing the beat frequency information between the transmitted and reflected signals, making it particularly suitable for short-range, high-precision, and highly dynamic measurement scenarios. For measuring blade tip distances in coaxial twin-rotor helicopters, millimeter-wave radar maintains stable ranging performance even under high relative velocity conditions. It operates independently of illumination and demonstrates strong adaptability to complex environments such as rain, fog, dust, and vibration [23]. Consequently, it serves as the core sensor for blade tip distance measurement during blade intersections.

The range resolution of FMCW radar is primarily determined by the modulation bandwidth [24]. Therefore, increasing the operating bandwidth of millimeter-wave radar can enhance range resolution capability, thereby improving ranging accuracy. In the application of measuring the blade tip distance of coaxial rotor helicopters, the radar must measure the instantaneous distance between observation points at the tips of the upper and lower rotors under conditions of high-speed rotor rotation. Since the overlap duration between upper and lower rotors is less than 1 ms, and electromagnetic waves propagate through the atmosphere at near-light speeds, the round-trip propagation time for the measurement signal is approximately 6.6 ns at the maximum tip distance of 990 mm. This duration is significantly shorter than the rotor overlap time window, satisfying the timing requirements for dynamic measurements. Therefore, by utilizing the phase information provided by the encoder, the millimeter-wave radar can be triggered to complete the measurement precisely at the moment of rotor intersection. This enables the transmission of signals, reception of echoes, and data acquisition within the limited time window.

**Figure 6:**
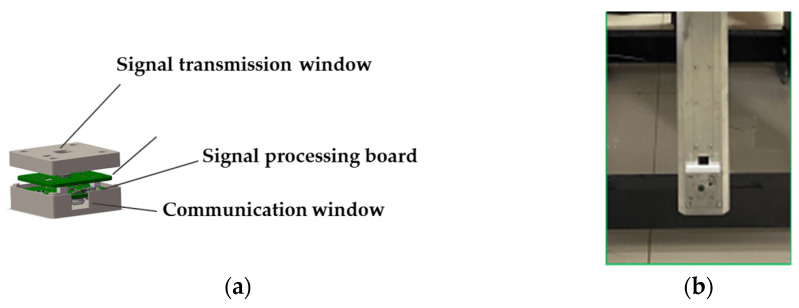
Millimeter-wave radar system: (**a**) schematic diagram of the enclosure; (**b**) embedded radar system within the rotating blade.

Based on the measurement requirements analysis and considering the volume, weight, and installation constraints imposed by coaxial helicopter platforms, this paper selects the TRA_120_045 millimeter-wave radar front-end module developed by Silicon Radar. Operating at a center frequency of 120 GHz with a modulation bandwidth of 20 GHz, it achieves a theoretical range resolution of 7.5 mm and covers a measurement range of 100–5000 mm, meeting the accuracy and range requirements for blade tip distance measurement. The FMCW radar module features an integrated encapsulation design. An antenna aperture is reserved on the top for electromagnetic wave transmission and reception, while communication interfaces on the side enable power supply and data transmission. As shown in Figure 6, the encapsulated module measures 44 mm × 42 mm × 18 mm and can be fully embedded into the upper surface of the lower rotor blade. Radar signal cables traverse pre-embedded conduits within the rotor and transmit data via slip rings to the ground signal processing terminal. At the moment of blade intersection, the millimeter-wave radar initiates measurement via a phase-triggered signal. Processing the returned echoes yields the instantaneous distance between the upper and lower rotor blade tips, providing the critical distance information for the multi-sensor fusion measurement system.

## 4. Measurement Principles and Theoretical Derivation of Blade Collision Warning Parameters

To address the potential collision risk between the upper and lower rotor blades in coaxial rotor helicopters, the rotor blade collision warning parameter d is adopted as the comprehensive evaluation parameter. The real-time blade collision warning parameter obtained by the measurement system is transmitted to the helicopter’s flight control system. The flight control system assesses the risk based on the parameter value and its trend. When the risk is high, it issues timely warnings and adjusts flight parameters to prevent blade collision accidents, thereby enhancing the safety performance of coaxial rotor helicopters. Therefore, in situ real-time measurement of the blade collision warning parameter d is critical, requiring measurement to be completed while the blades are in high-speed dynamic motion.

### 4.1. Principle of Blade Collision Warning Parameter Measurement

To measure blade collision warning parameters, it is necessary to directly or indirectly measure the positions of all the upper and lower blade tip observation points at the moment of blade intersection. The measurement challenges lie in the following three aspects: First, the blade tips are in high-speed dynamic motion, with the upper and lower rotor blades passing each other in less than 1 millisecond and adjacent passes occurring within 20 milliseconds. Measurement signal acquisition must be completed within the blade intersection time, and rapid measurements must be performed between adjacent passes. Second, both the tip distance and tip position exhibit time-varying characteristics. The two tip observation points defining the blade tip distance are both in high-speed motion. Furthermore, the individual flapping and oscillating motions of the blades cause the tip observation points to exhibit high-speed tangential movement with continuously varying relative coaxial positions. This results in the blade tip distances at each intersection point not only varying randomly in magnitude but also lacking a unified positional reference. This places extremely high demands on the measurement system, necessitating the establishment of a unified coordinate reference for the tip positions. Third, the blade collision warning parameter measurement system must support in situ real-time measurement. When developing measurement methodologies, actual helicopter flight conditions must be considered to ensure the system can transition from laboratory validation to in-flight testing.

**Figure 7:**
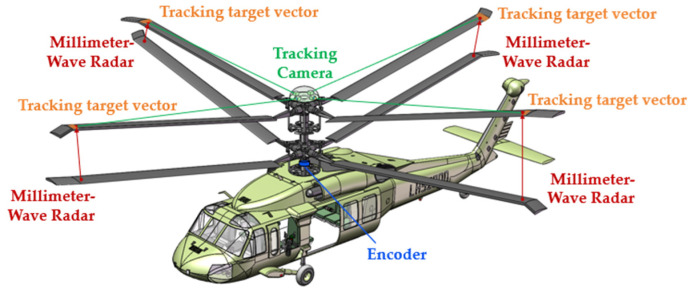
Blade collision warning parameter measurement method.

Considering the applicability of various measurement methods, the proposed blade collision warning parameter measurement method is illustrated in Figure 7. Blade intersection information is obtained through phase measurement. By installing an angle measurement device at an appropriate location on the drive shaft transmission chain and determining the phase zero point relative to the airframe, each intersection phase becomes fixed and measurable. The upper blade tip observation point location is determined through a tracking visual positioning method. A visual measurement device consisting of four tracking cameras is installed at the upper rotor hub. The device is designed with a fairing fixed to the upper rotor shaft end, enabling synchronous rotation with the upper rotor system. A tracking target vector is mounted at the upper blade tip observation point as a marker, enabling the spatial position of the observation point relative to the tracking cameras to be determined. The lower blade tip observation point position is indirectly obtained by measuring the distance between the upper and lower blade tips. A distance measurement device is installed at the lower blade tip observation point to measure the distance between the blade tips at the moment of intersection. Combined with the corresponding upper blade tip observation point position, the lower blade tip observation point position can be calculated.

After completing measurements of the upper and lower blade tips at the intersection point, the upper and lower limits of the rotor structure cone are constructed using the lowest point of the upper blade tip and the highest point of the lower blade tip within a unified coordinate system. The blade collision warning parameters are calculated and the measured values outputted from this measurement before the next blade intersection occurs.

### 4.2. Construction of the Coaxial Twin-Rotor Coordinate Measurement Model

When constructing a measurement coordinate model, it must align with the motion characteristics of the rotor and the measurement characteristics of the parameters being measured. In a coaxial twin-rotor helicopter, the upper and lower rotors rotate at the same speed but in opposite directions. Assuming the lower rotor rotates counterclockwise with rotational speed ω and rotation angle θ, the upper rotor rotates clockwise with rotational speed −ω and rotation angle −θ. The observation points at the tips of the upper rotor blades are A, C, E, and G, while those at the tips of the lower rotor blades are B, D, F, K. When the blades are undeformed, the distance from each observation point to its corresponding blade root center is equal, denoted as R. The positions of M_A_, M_C_, M_E_, M_G_ represent the upper blade root centers, while M_B_, M_D_, M_F_, M_K_ denote the lower blade root centers. According to rotor structural design principles, blade deformation primarily occurs at the root. Deformation from the root to the rotor shaft during rotation is neglected here. The integrated coordinate system comprises the airframe coordinate system, lower rotor coordinate system, upper rotor coordinate system, camera coordinate system, and attitude sensor coordinate system.

**Figure 8:**
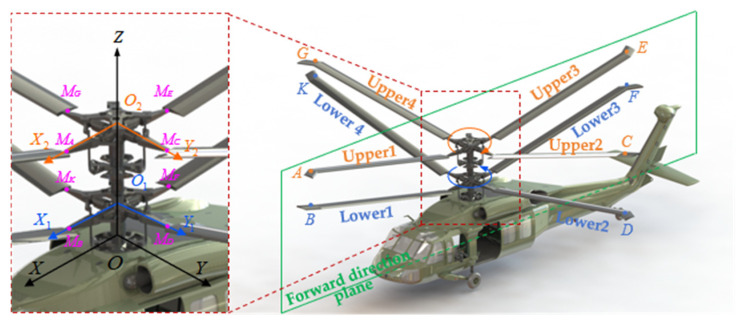
Coaxial twin-rotor helicopter fuselage and rotor coordinate system.

First, the fuselage coordinate system, lower rotor coordinate system, and upper rotor coordinate system are established separately. As shown in Figure 8, the fuselage coordinate system is O-XYZ, where O is the intersection point of the rotor axis and the fuselage, OZ is the direction of the rotor axis, OX lies in the forward flight plane and is perpendicular to the rotor axis, and OY is perpendicular to the forward flight plane. The lower rotor coordinate system is $O_{1}-X_{1}Y_{1}Z$, where $O_{1}$ is the center of the lower rotor hub. The $O_{1}X_{1}$ axis lies in the plane formed by observation point B on lower blade 1 and the OZ axis and is perpendicular to the rotor axis. The $O_{1}Y_{1}$ axis is perpendicular to both the $O_{1}X_{1}$ axis and the OZ axis. The upper rotor coordinate system is $O_{2}-X_{2}Y_{2}Z$, where $O_{2}$ is the center of the upper rotor hub. $O_{2}X_{2}$ lies in the plane formed by observation point A on upper blade 1 and OZ and is perpendicular to the rotor axis. $O_{2}Y_{2}$ is perpendicular to both $O_{2}X_{2}$ and OZ. The distance between the hub centers of the upper and lower rotors is $O_{1}O_{2}=H_{0}$. The distance from the hub center of the lower rotor to the intersection point of the rotor axis and the airframe is $OO_{1}=l$.

Assuming the coordinates of an observation point in the lower rotor coordinate system are $\left(x_{1},y_{1},z_{1}\right)$, and its coordinates in the airframe coordinate system are $\left(x,y,z\right)$, then the transformation relationship between the lower rotor coordinate system $O_{1}-X_{1}Y_{1}Z$ and the airframe coordinate system O-XYZ is

(2) $${\left[\begin{array}{cccc} x & y & z & 1 \end{array}\right]}^{T}=T_{1}R_{1}{\left[\begin{array}{cccc} x_{1} & y_{1} & z_{1} & 1 \end{array}\right]}^{T},$$

The homogeneous transformation matrix $R_{1}$ and translation matrix $T_{1}$ are respectively

(3) $$R_{1}=\left[\begin{array}{cccc} \cos\theta & -\sin\theta & 0 & 0\\ \sin\theta & \cos\theta & 0 & 0\\ 0 & 0 & 1 & 0\\ 0 & 0 & 0 & 1 \end{array}\right],$$

(4) $$T_{1}=\left[\begin{array}{cccc} 1 & 0 & 0 & 0\\ 0 & 1 & 0 & 0\\ 0 & 0 & 1 & l\\ 0 & 0 & 0 & 1 \end{array}\right],$$

Assuming the coordinates of an observation point in the upper rotor coordinate system are $\left(x_{2},y_{2},z_{2}\right)$, and its coordinates in the fuselage coordinate system are $\left(x,y,z\right)$, then the transformation relationship between the upper rotor coordinate system $O_{2}-X_{2}Y_{2}Z$ and the fuselage coordinate system O-XYZ is

(5) $${\left[\begin{array}{cccc} x & y & z & 1 \end{array}\right]}^{T}=T_{2}R_{2}{\left[\begin{array}{cccc} x_{2} & y_{2} & z_{2} & 1 \end{array}\right]}^{T},$$

The homogeneous transformation matrix $R_{2}$ and translation matrix $T_{2}$ are respectively

(6) $$R_{2}=\left[\begin{array}{cccc} \cos\theta & \sin\theta & 0 & 0\\ -\sin\theta & \cos\theta & 0 & 0\\ 0 & 0 & 1 & 0\\ 0 & 0 & 0 & 1 \end{array}\right],$$

(7) $$T_{1}=\left[\begin{array}{cccc} 1 & 0 & 0 & 0\\ 0 & 1 & 0 & 0\\ 0 & 0 & 1 & \left(l+H_{0}\right)\\ 0 & 0 & 0 & 1 \end{array}\right],$$

Therefore, the coordinates of the observation point at the rotor blade tip can be measured in the corresponding rotor coordinate system and then converted into coordinates in the airframe coordinate system.

**Figure 9:**
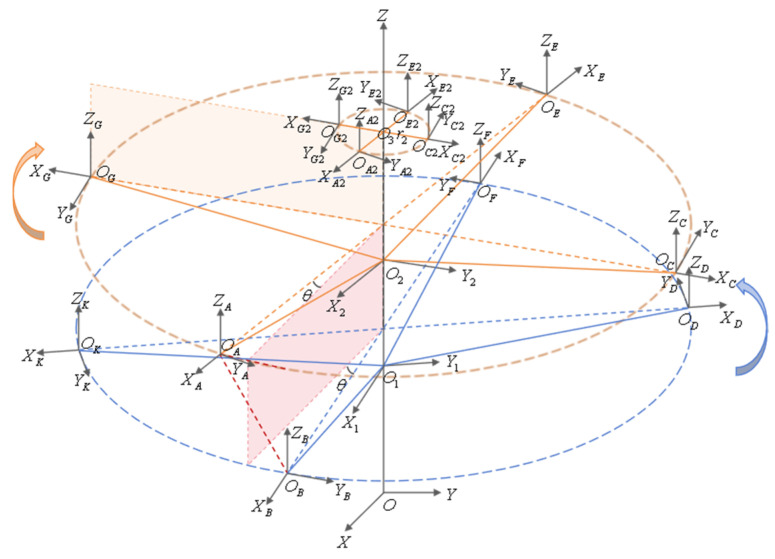
Coaxial twin-rotor coordinate measurement model.

Then, the follow-up camera coordinate system and the attitude sensor coordinate system are established. The tracking cameras of the visual measurement system are mounted above the upper rotor hub and rotated with the upper rotor, maintaining relative stillness with respect to the hub. Attitude sensors can be installed at observation points on the upper and lower blade tips as required to monitor changes in blade attitude, providing auxiliary measurements of tip motion characteristics. As shown in Figure 9, after establishing the airframe coordinate system, lower rotor coordinate system, and upper rotor coordinate system, the tracking camera coordinate systems are designated as $O_{A2}-X_{A2}Y_{A2}Z_{A2}$, $O_{C2}-X_{C2}Y_{C2}Z_{C2}$, $O_{E2}-X_{E2}Y_{E2}Z_{E2}$, and $O_{G2}-X_{G2}Y_{G2}Z_{G2}$, respectively. The attitude sensor coordinate systems are labeled as $O_{A}-X_{A}Y_{A}Z_{A}$, $O_{C}-X_{C}Y_{C}Z_{C}$, $O_{E}-X_{E}Y_{E}Z_{E}$, $O_{G}-X_{G}Y_{G}Z_{G}$, $O_{B}-X_{B}Y_{B}Z_{B}$, $O_{D}-X_{D}Y_{D}Z_{D}$, $O_{F}-X_{F}Y_{F}Z_{F}$, and $O_{K}-X_{K}Y_{K}Z_{K}$. The distance from the center of the upper rotor hub to the center of the visual measurement device is $O_{2}O_{3}=l_{2}$. The origins of the tracking camera coordinate systems $O_{A2}$, $O_{C2}$, $O_{E2}$, and $O_{G2}$ to the center of the visual measurement device is $r_{2}$, i.e., $O_{3}O_{A2}=O_{3}O_{C2}=O_{3}O_{E2}=O_{3}O_{G2}=r_{2}$.

Taking observation point C at the tip of the rotor blade as an example, its coordinates in the follow-up camera coordinate system $O_{2C}-X_{2C}Y_{2C}Z_{2C}$ are $\left({x_{C}}^{\prime },{y_{C}}^{\prime },{z_{C}}^{\prime }\right)$. After first transforming these coordinates to the upper rotor coordinate system and then to the airframe coordinate system, the resulting coordinates are $\left(x_{C},y_{C},z_{C}\right)$. The transformation relationship is

(8) $${\left[\begin{array}{cccc} x_{C} & y_{C} & z_{C} & 1 \end{array}\right]}^{T}=T_{2}R_{2}T_{C}R_{C}{\left[\begin{array}{cccc} {x_{C}}^{\prime } & {y_{C}}^{\prime } & {z_{C}}^{\prime } & 1 \end{array}\right]}^{T},$$

The homogeneous transformation matrix $R_{C}$ and translation matrix $T_{C}$ are respectively

(9) $$R_{C}=\left[\begin{array}{cccc} \cos\left(270^{\circ }\right) & \sin\left(270^{\circ }\right) & 0 & 0\\ -\sin\left(270^{\circ }\right) & \cos\left(270^{\circ }\right) & 0 & 0\\ 0 & 0 & 1 & 0\\ 0 & 0 & 0 & 1 \end{array}\right],$$

(10) $$T_{C}=\left[\begin{array}{cccc} 1 & 0 & 0 & r_{2}\\ 0 & 1 & 0 & 0\\ 0 & 0 & 1 & l_{2}\\ 0 & 0 & 0 & 1 \end{array}\right],$$

The transformation from the respective camera coordinate systems to the aircraft coordinate system for observation points A, E, and G can be derived similarly.

The attitude sensor coordinate system is a dynamically changing coordinate system that provides the tip attitude information for the measurement system. Taking observation point A as an example, the calibration of the positional relationship between the attitude sensor coordinate system $O_{A}-X_{A}Y_{A}Z_{A}$ and the airframe coordinate system must be completed prior to measurement. During motion, the attitude sensor can perceive its own velocity, acceleration, and angular information, thereby inferring changes in the rotation matrix to obtain the real-time tip attitude angle $\left(\Psi_{Z-A},\theta_{X-A},\phi_{Y-A}\right)$, where $\Psi_{Z-A}$, $\theta_{X-A}$, and $\phi_{Y-A}$ represent the rotation angles about the $Z_{A}$, $X_{A}$, and $Y_{A}$ axes, respectively. The attitude angles for other observation points can be obtained similarly. This completes the construction of the coaxial twin-rotor measurement coordinate model.

### 4.3. Calculation Framework for Blade Collision Warning Parameters

**Figure 10:**
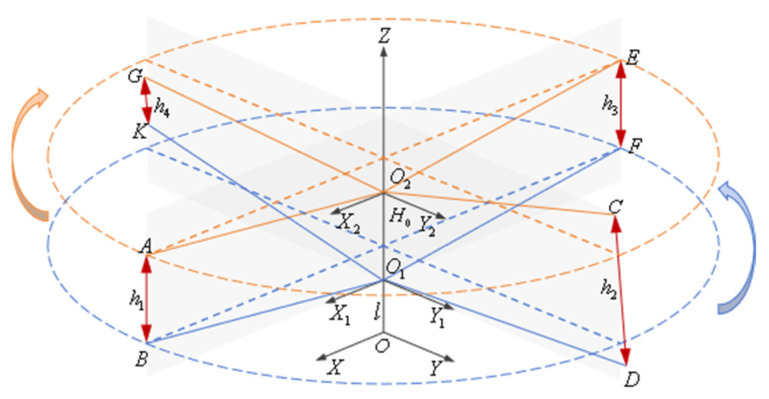
Measurement parameter specifications for a certain intersection moment.

**Figure 11:**
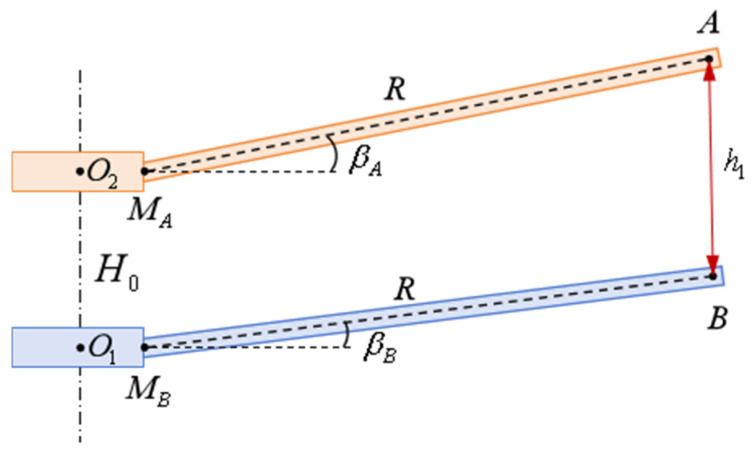
Schematic diagram of blade tip observation point position calculation at a certain intersection moment.

The calculation of blade collision warning parameters is based on the blade intersection state, with each intersection yielding a parameter value. Taking an eight-bladed coaxial twin-rotor helicopter as an example, the blades intersect every $45^{\circ }$. The intersecting blades between the upper and lower rotors can be determined by the rotation angle θ. When θ=0, the intersecting blades are A-B, C-D, E-F, and G-K. When $\theta =45^{\circ }$, the intersecting blades are A-K, C-B, E-D, G-F; when $\theta =90^{\circ }$, the intersecting blades are A-F, C-K, E-B, G-D; when $\theta =135^{\circ }$, the intersecting blades are A-D, C-F, E-K, G-B. When the rotation angle differs by $n\cdot 180^{\circ }\left(n=1,2,\ldots \right)$, the intersecting blades are identical. As shown in Figure 10, using this intersection state as an example for measurement parameter explanation, the observation points A, C, E, and G, measured by the visual measurement device, are converted to the coordinate system O-XYZ, with coordinates $\left(x_{A},y_{A},z_{A}\right)$, $\left(x_{C},y_{C},z_{C}\right)$, $\left(x_{E},y_{E},z_{E}\right)$, and $\left(x_{G},y_{G},z_{G}\right)$, respectively. The distances measured by the device to the blade tips are $h_{1}$, $h_{2}$, $h_{3}$, and $h_{4}$, respectively. The position of the lower blade tip observation point requires calculation based on the upper blade tip position, blade tip distance, and swing angle. As shown in Figure 11, $M_{A}$ is the center point of the upper blade root, $M_{B}$ is the center point of the lower blade root, $M_{A}A=M_{B}B=R$, while $\beta_{A}$ and $\beta_{B}$ are the swing angles of the upper and lower blades, respectively. Let the coordinates of the lower blade tip observation point B in the O-XYZ coordinate system be $\left(x_{B},y_{B},z_{B}\right)$. Then, the Z-axis coordinate value of point B is

(11) $$z_{B}=z_{A}-h_{1}\cdot \cos\left[\arcsin\left(\frac{R\left(\cos\beta_{B}-\cos\beta_{A}\right)}{H_{0}}\right)\right].$$

The coordinate values of observation points D, F, and K on the lower blade tip along the Z-axis can similarly be obtained as $Z_{D}$, $Z_{F}$, and $Z_{K}$, respectively. In practical applications, to reduce the number of sensor modules, the calculation of the observation point positions on the lower blade tip can be simplified to

(12) $$z_{B}\approx z_{A}-h_{1},$$

**Figure 12:**
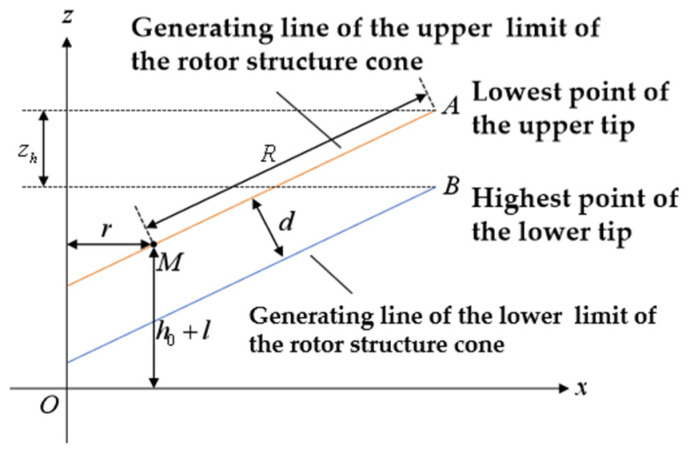
Construction of upper and lower limits of the rotor structure cone.

The construction of the upper and lower limit conical surfaces is illustrated in Figure 12. After obtaining the positions of the upper and lower blade tips, the minimum Z-direction values of tips A, C, E, and G are taken as $\min\left\{Z_{A},Z_{C},Z_{E},Z_{G}\right\}$, while the maximum Z-direction values of tips B, D, F, and K are taken as $\max\left\{Z_{B},Z_{D},Z_{F},Z_{K}\right\}$. These values are combined with the blade root center point M to construct the upper and lower limit conical surfaces. In the xOz coordinate system, the generatrix of the upper limit construction cone is the line connecting the lowest point of the upper blade tip and the blade root center point. Its generatrix equation is $z=k\left(x-r\right)+H_{0}+l$, where the slope k depends on the coordinates of the lowest point of the upper blade tip, and $k=\tan\left[\arcsin\left(\min\left\{Z_{A},Z_{C},Z_{E},Z_{G}\right\}-H_{0}-l\right)/R\right]$; the generatrix of the lower limit construction cone is parallel to the upper limit construction cone and passes through the highest point of the lower blade tip, with the equation $z=k\left(x-r\right)+H_{0}+l-z_{h}$, where $z_{h}$ is the distance between the projections of the lowest point of the upper blade tip and the highest point of the lower blade tip onto the common axis.

After constructing the upper and lower limits of the rotor structure cone, the distance d between these two limit surfaces serves as the blade collision warning parameter. This parameter can be used as a comprehensive expression for the danger proximity warning, with its formula as follows:

(13) $$d=\frac{z_{h}}{\sqrt{1+k^{2}}}=\frac{\min\left\{Z_{A},Z_{C},Z_{E},Z_{G}\right\}-\max\left\{Z_{B},Z_{D},Z_{F},Z_{K}\right\}}{\sqrt{1+k^{2}}},$$

Clearly, the value of d depends on the distance factor h between the tips of the upper and lower rotor blades, the position factor k, and the rotor intersection phase. It fully accounts for the possibility of phase differences between the lowest point of the upper rotor blade tip and the highest point of the lower rotor blade tip. Therefore, the blade collision warning parameter d serves as a real-time effective parameter for blade collision warning in coaxial twin-rotor systems.

In practical applications, real-time monitoring of the blade collision warning parameter d and tracking its trend enable prediction of potential hazardous situations. To allow sufficient time for the helicopter to adjust its flight state in response to impending hazards, an appropriate warning threshold $d_{th}$ must be selected based on the helicopter model. When $d>d_{th}$, the system remains in a safe state. Conversely, if $d\leq d_{th}$, a warning signal is transmitted to the flight control system, prompting timely adjustment of flight parameters to establish closed-loop control and ensure safe helicopter operation.

## 5. Experimental Validation and Measurement Performance Evaluation

To evaluate the proposed measurement method in a problem-oriented manner, the experimental validation in this section is organized around the following questions: (1) whether the blade collision warning parameter can be measured with sufficient accuracy; (2) whether the measurement results are repeatable under identical operating conditions; (3) whether the proposed system maintains valid measurement performance under dynamic crossing conditions over the tested rotational-speed range. The following experimental results are therefore interpreted with respect to these three aspects: accuracy, repeatability, and dynamic applicability.

### 5.1. Dynamic Experimental Platform

To effectively simulate the coaxial twin-rotor blade intersection process in a laboratory environment and validate the dynamic applicability of the proposed blade collision warning parameter measurement method, this paper constructed a rotor motion simulation experimental platform. The platform design aims to achieve adjustable and measurable blade tip distance, determinable blade intersection phase, and controllable rotational speed, thereby meeting the experimental requirements for multi-sensor dynamic measurement and performance evaluation.

**Figure 13:**
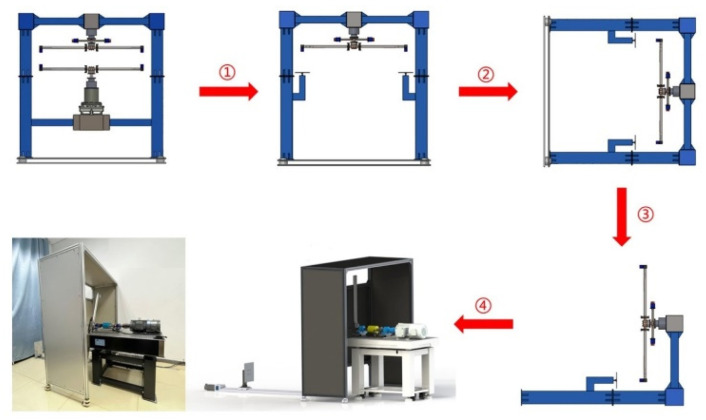
Iterative design of the dynamic experimental platform.

**Figure 14:**
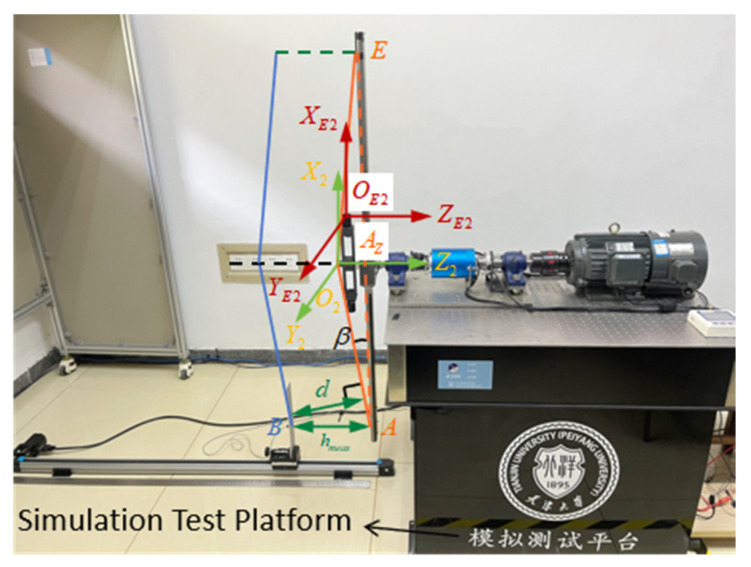
Physical diagram of dynamic measurement experimental setup.

As shown in Figure 13, during the initial design phase, the platform adopted a split dual-drive structure to achieve relative motion between the upper and lower blades, incorporating an adjustable mechanism to introduce a reference value for blade tip distance. However, further analysis revealed that multi-drive systems struggle to maintain stable and consistent intersection phases between upper and lower blades under dynamic operating conditions, adversely affecting the determinism of phase-related measurement parameters. Given this constraint, the platform structure was modified to a single-side rotation configuration. By driving only one side of the blades to rotate while keeping the opposite side stationary, the moment of blade intersection is uniquely determined by the angular position of the rotating blade. This significantly enhances the determinacy of the phase measurements. Furthermore, to balance laboratory space constraints and safety during high-speed rotation, the overall platform layout is modified from a vertical to a horizontal orientation. This positioning of the rotation plane in the vertical direction reduces the hazardous area generated by high-speed rotation, enhancing both operational safety and manageability. Through continuous simplification and optimization of structural form and functional modules, a horizontal, split-type, single-side rotation experimental platform suitable for long-term laboratory operation is ultimately developed. The physical configuration is illustrated in Figure 14.

It should be noted that the present single-side rotation platform is intended to provide a representative validation environment for the key measurement chain of the proposed measurement method, rather than to fully reproduce the complete aeromechanical state of a real coaxial counter-rotating rotor system. For the measurement task considered in this paper, the key validation requirements are a repeatable blade intersection event with deterministic phase, a controllable instantaneous tip distance at the intersection location, and a sufficiently short acquisition window for synchronized phase, position, and distance measurement. These requirements can be reproduced on the present platform by fixing one blade at the nominal intersection position and driving the other blade to sweep through the sensing region at a controlled rotational speed. As shown in Table 1, the platform covers the principal temporal and geometric constraints relevant to the measurement chain, including blade tip distance range, overlap duration, rotor speed range, and the interval between adjacent intersections. Therefore, the platform is functionally equivalent for validating phase-triggered acquisition, multi-sensor fusion, and warning parameter reconstruction under short duration dynamic intersection conditions.

Nevertheless, this platform is not fully equivalent to an actual dual-rotor counter-rotating helicopter in the aeromechanical sense. It does not reproduce the simultaneous motion of both rotors, mutual aerodynamic interference, blade deformation, hub-coupled vibration, or phase fluctuations under real flight loads. Therefore, the present experiments verify the feasibility and measurement performance of the proposed method under representative dynamic intersection conditions. Further validation is still required on the twin-rotor test platform and under conditions that more closely resemble in-flight loads.

### 5.2. Experimental System Overview

**Figure 15:**
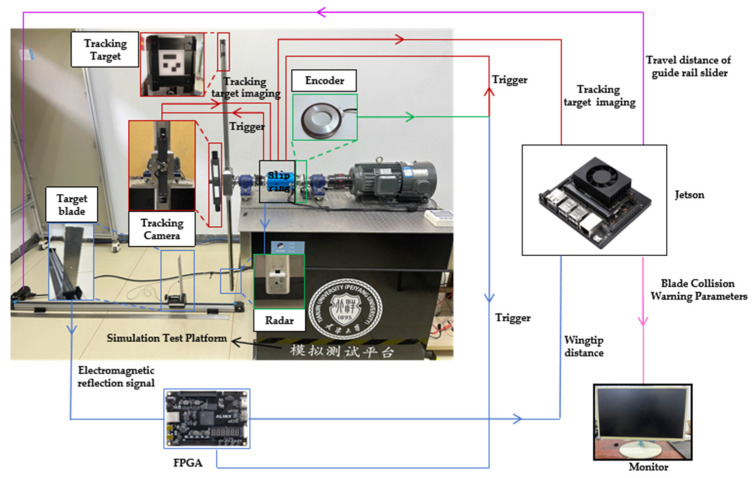
Blade collision parameter measurement system based on experimental platform.

To validate the feasibility and measurement performance of the proposed blade collision warning parameter measurement method under representative dynamic intersection conditions provided by the simulation platform, this paper constructs a multi-sensor dynamic measurement experimental system based on encoders, a tracking camera, and millimeter-wave radar. As shown in Figure 15, this system achieves synchronous measurement of blade motion phase, tip spatial position, and tip-to-tip distance at the moment of intersection through the collaborative acquisition and fusion processing of multi-source information. This provides an experimental foundation for analyzing the evolution characteristics of tip-to-tip distance under dynamic operating conditions.

In the experimental system, an encoder is mounted on the rotating drive shaft end to capture real-time blade rotation angle data, serving as the reference for blade phase determination and time synchronization. The tracking camera maintains alignment with the blade’s motion direction, employing visual measurement methods to capture the spatial position of the blade tip within the camera coordinate system. Millimeter-wave radar is positioned at the blade tip to directly measure the blade tip distance between the upper and lower blades during their intersection. The data from all sensors undergo fusion through a unified time synchronization mechanism and spatial mapping model, establishing a unified representation of blade tip motion parameters and the minimum blade tip distance.

The blade collision parameter measurement system is developed based on the Jetson architecture. It implements tip distance measurement on the millimeter-wave radar integrated module and performs real-time image processing and calculates blade collision warning parameters on the Jetson platform as the host computer. Additionally, Qt is utilized to complete the software design of the measurement system control panel, as well as the design of the data recording and saving functions.

The radar can receive trigger signals from the Jetson platform, transmit electromagnetic signals, process the received signals to obtain the blade tip distance value for each intersection, and send it to the Jetson platform.

Regarding the processing of blade tip distance within the radar ranging module, a phase-based filtering approach is employed. When multiple distance values are obtained for a set of passing blades during a single pass, the distance value with the phase closest to $n\times 45^{\circ }\left(n=0,1,\ldots ,7\right)$ is selected as the tip distance measurement result and transmitted to the Jetson platform. Here, the value of n depends on the theoretical phase of the passing blades. For this simulation experimental platform, a blade intersection occurs when the radar and the measured blade are directly aligned, meaning n is set to 0.

The Jetson-based blade collision warning parameter measurement system processes images captured by a tracking camera after triggering an emission signal to obtain tip position data. It then calculates collision warning parameters by integrating the tip distance information. The system also features the capability to alter measurement conditions during operation by adjusting the receiver transducer’s position through controlled movement of the guide rail slider. To ensure real-time calculation of blade collision warning parameters for the limit cone distance measurement system, the NVIDIA Jetson Xavier NX development platform was selected for system construction and development. This embedded device offers multi-threading capabilities, real-time image processing, and robust computational power. Adopting object-oriented programming principles, the system functionalities are modularized. The main thread maintains continuous system operation, while the sub-threads include image acquisition, image processing, Ethernet reception, guide rail control, and blade collision warning parameter calculation. The image acquisition and processing thread implements automatic tip position measurement. The tracking camera captures images via external triggering, feeding photos of the tracking target vector into the image processing module. Raw images undergo preprocessing through image filtering and histogram equalization functions, followed by tip coordinate extraction using the ArUco positioning algorithm. The Ethernet reception thread establishes communication with the radar module, receiving and storing blade tip distance calculations. The guide rail control thread enables real-time adjustment of blade tip distance. The limit cone distance calculation thread integrates the blade tip position and distance calculations. It sets the coordinates of the blade tip position and the corresponding distance value as a data set. It then calculates the position of each blade tip in the same coordinate system during a single blade intersection. This enables the calculation of the blade collision warning parameters for that intersection.

The measurement system enables real-time monitoring of blade collision warning parameters, requiring performance verification through comparison with reference values. The blade collision warning parameters are calculated from the tip distance and tip position during blade overlap. Consequently, their reference values can also be derived from the reference values for the tip distance and tip position. The reference tip distance is provided by the guide rail, while the reference tip position is determined by the calibration value during installation, thereby yielding the reference values for the blade collision parameters. It should be noted that during actual blade motion, flapping, flutter, and torsional movements cause changes in tip position. On the simulation experimental platform, to validate the collision warning parameter measurement system’s functionality in dynamic conditions while providing tip position reference values and considering safety factors, the simulated blade is structurally designed to ensure rigidity during high-speed operation. This ensures that the deformation of the simulated blade tip is negligible compared to the measurement error metrics, allowing the initial blade tip position to serve as the reference value.

**Figure 16:**
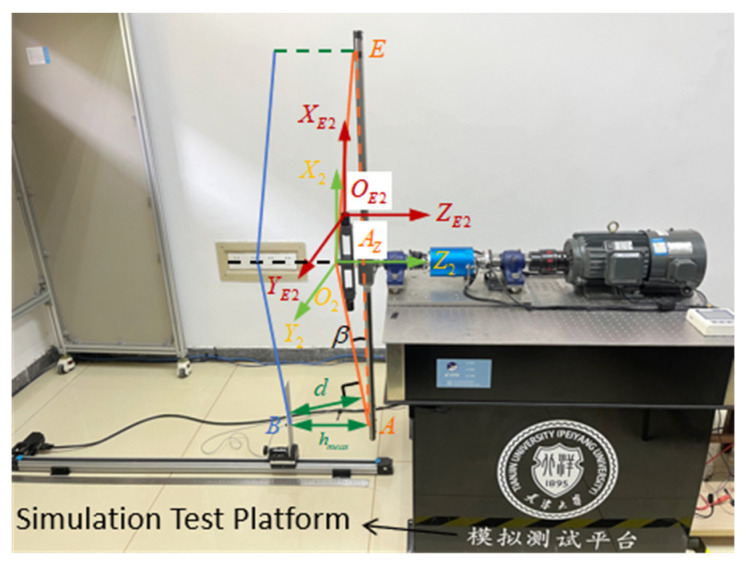
Rotor coordinate model based on experimental platform.

The rotor coordinate model from Section 4.2 is adopted. The rotor coordinate definition based on the experimental platform is shown in Figure 16. In this coordinate system, $O_{2}-X_{2}Y_{2}Z_{2}$ represents the vertical upward direction, with point $O_{2}$ being the shaft end center point. The $O_{2}X_{2}$ direction corresponds to the shaft axis, while $O_{2}Y_{2}$ is perpendicular to both $O_{2}X_{2}$ and $O_{2}Z_{2}$. In the coordinate system $O_{E2}-X_{E2}Y_{E2}Z_{E2}$, $O_{E2}$ represents the optical center of the camera, with $O_{E2}X_{E2}$, $O_{E2}Y_{E2}$, and $O_{E2}Z_{E2}$ parallel to the $O_{2}X_{2}$, $O_{2}Y_{2}$, and $O_{2}Z_{2}$ axes, respectively. Point A is the surface center of the transmitter transducer, point B is the surface center of the receiver transducer, and point E is the surface center of the tracking target vector. The intersection of AE and $O_{2}Z_{2}$ is $A_{Z}$. One tracking camera captures the image of the tracking target vector, while the other serves as a counterweight. The vertical distance $h_{meas}$ between the transmitter and receiver transducers is measured by the blade tip distance measurement module. Since the simulation experimental platform was not designed to simulate the rotor blade flapping motion, the simulated rotor blade tip position lies in the same plane as the hub. This weakens the effect of the limiting cone surface. To visually illustrate the limiting construction cone surface, the line connecting the fixed point $O_{2}$ at the shaft end center and the observation point A serves as the generatrix of the upper limit construction cone surface. The cone surface with axis $O_{2}Z_{2}$ constitutes the upper limit construction cone surface. The plane passing through point B and parallel to the upper limit construction cone surface forms the lower limit construction cone surface. Angle β serves as the pre-cone angle, calculated from measurements taken at the follow-up camera installation calibration position and the blade tip position module. The specific process is as follows: The coordinate position difference between $O_{2}$ and $O_{E2}$ in the $O_{2}Z_{2}$ direction is Δz, and the coordinate position difference in the $O_{2}X_{2}$ direction is Δx. The measured value of point E in the coordinate system $O_{E2}-X_{E2}Y_{E2}Z_{E2}$ is $\left(x_{E2},y_{E2},z_{E2}\right)$. Then, the construction swing angle $\beta =\arctan\left(\left(\Delta z+z_{E2}\right)/\left(\Delta x+x_{E2}\right)\right)$. On the simulation experimental platform, since only one set of measurement modules is used for measurement, one tip distance value and one upper tip position value are obtained. Here, A and E are treated as two points, symmetrical about the rotation axis. Thus, E is taken as the lowest observation point on the upper blade, and B as the highest point of the lower blade observation point. Therefore, the blade collision warning parameter is $d=h_{meas}\cdot \cos\beta$.

### 5.3. Measurement Process and Results

The measurement procedure is as follows.

Step 1: Installation.

Install the blade collision parameter measurement system on the simulation platform and verify its secure mounting.

Step 2: Initial Positioning and Calibration.

Adjust the position of the test blade via the guide rail to align its surface with the rotating blade surface; adjust the tracking camera’s focal length and aperture to calibrate its intrinsic parameters; calibrate the tracking camera’s Δx and Δz mounting positions.

Step 3: Measurement.

Power on; start the motor, adjust its speed to 420 r/min, and lock the motor speed; use the Jetson platform to adjust the position of the tested blade, changing the blade-to-blade distance $l_{R}$. Each adjustment of 100 mm constitutes one measurement point, with an adjustment range of 99–990 mm, totaling 10 measurement points.

Note: To ensure stable operation of the simulated blade, maintain its running state unchanged when switching measurement points, thereby guaranteeing measurement consistency.

Step 4: Complete measurement and export data.

The results of a single experimental measurement are shown in Table 2. The parameters in Table 3 are intermediate variables used to construct the collision risk parameters.

Analysis of the data in the table reveals that due to the sufficient rigidity of the simulated rotor, the deformation during high-speed rotation is minimal. Consequently, the coordinates of the tracking target vector calculated from tip position measurements remain virtually unchanged in the camera coordinate system. By integrating tip distance measurements with tip position measurements and the calibrated installation position of the tracking camera, the measured values and measurement errors of the blade collision warning parameters can be calculated. These are used to assess collision risks between the upper and lower rotors, thereby validating the measurement capabilities of the blade collision warning parameter measurement system.

**Figure 17:**
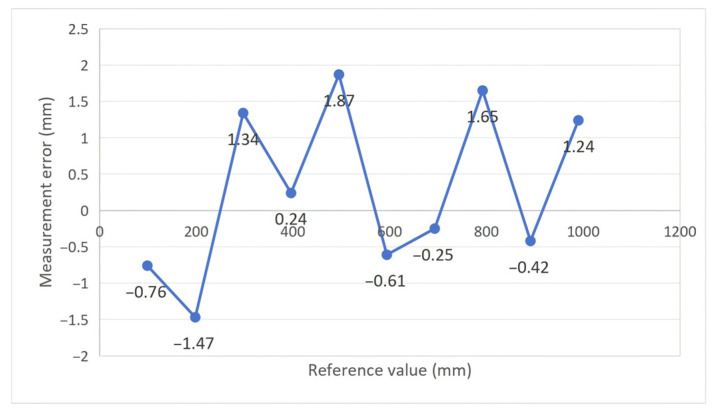
Distribution of measurement errors in collision warning parameters.

Accuracy validation. As shown in Figure 17, the measurement error of the blade collision warning parameter exhibits a relatively stable distribution characteristic across the entire measurement range. As the reference blade tip distance increases, the overall error fluctuates around zero, without exhibiting significant systematic bias or trend toward range amplification. The maximum positive deviation is 1.87 mm, while the maximum negative deviation is −1.47 mm. Throughout the full range, error amplitudes remain significantly below the 15 mm engineering requirement specified in the introduction, demonstrating the high measurement accuracy and reliability of the proposed method. The error distribution characteristics reveal consistent variation across the measurement points, with no isolated anomalies or abrupt changes. This confirms that under dynamic rotor conditions, the multi-sensor fusion model stably tracks blade tip position evolution and effectively estimates the minimum tip distance at the blade intersection. These results validate the applicability of the proposed blade collision warning parameter and its measurement method under varying tip distance conditions, providing reliable experimental evidence for subsequent blade collision warning assessment and real-time early warning systems. These results verify that the proposed method achieves satisfactory measurement accuracy under the tested laboratory conditions.

Repeatability validation. At each designated measurement point, multiple sets of blade collision warning parameter measurements are obtained by simulating continuous rotation of the blades over multiple revolutions. Since all measurements are conducted under identical operating conditions and measurement states, these results serve as repeatability test data. The mean and standard deviation of the repeatability test results across different measurement points are summarized in Table 4. Among the five repeatability experiments at different measurement points, the maximum standard deviation of the blade collision warning parameter measurements is 0.67 mm, corresponding to a maximum coefficient of variation of 0.11%. These results indicate that under identical measurement conditions, the proposed blade collision warning parameter measurement method exhibits good repeatability, with random measurement errors maintained at a low level. These results demonstrate that the proposed method provides good repeatability under identical operating conditions.

### 5.4. Measurement Performance at Different Rotational Speeds

Dynamic applicability across rotational speeds. To further evaluate the applicability of the proposed measurement method over the rotational speed range of the experimental platform, additional experiments are carried out at representative rotational speeds of (300, 420, 600, 900, and 1200) r/min. At each speed, the same measurement procedure described above is repeated under identical installation and calibration conditions. The reference blade tip distance is adjusted from 99 mm to 990 mm with an interval of approximately 100 mm, and the blade collision warning parameter is calculated for each measurement point.

**Figure 18:**
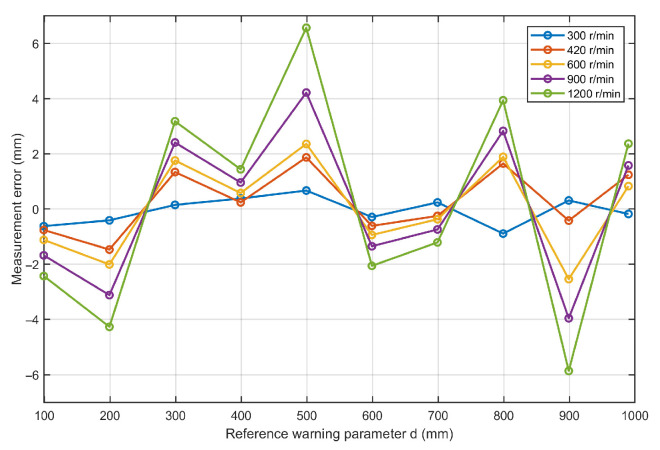
Measurement error of warning parameter at different rotational speeds.

The experimental results are summarized in Figure 18. As the rotational speed increases, the blade intersection duration decreases and the temporal requirement for synchronous phase, position, and distance acquisition becomes more stringent. Nevertheless, the proposed system maintains stable operation throughout the tested speed range. The measurement error of the blade collision warning parameter ranges from −5.86 mm to 6.57 mm, and the maximum absolute error at all tested speeds does not exceed 6.57 mm. These results indicate that the proposed method maintains satisfactory measurement accuracy over the tested rotational speed range, although the fluctuation of the results increases moderately at higher speeds.

A comparison of the results at different rotational speeds shows that the increase in speed did not lead to a significant degradation in measurement accuracy, although a moderate increase in fluctuation is observed at the highest tested speeds due to the shorter acquisition window and the stronger dynamic disturbance. Overall, the results indicate that the proposed method is applicable over the tested rotational speed range of the simulation platform and can maintain satisfactory accuracy and stability under representative dynamic conditions.

In addition to the experimentally validated speed range, the expected performance of the proposed method under other operating conditions can be qualitatively discussed as follows. As the rotor speed increases, the blade overlap duration becomes shorter, which reduces the available acquisition window for the synchronized phase, position, and distance measurement. Under this condition, the proposed phase-triggered architecture remains applicable, but the system becomes more sensitive to trigger latency and synchronization errors. Sensor misalignment mainly affects two stages of the measurement chain. Misalignment in the camera branch may introduce bias into the image-based tip localization and the subsequent coordinate mapping, while misalignment in the radar branch may reduce echo strength or shift the effective ranging direction away from the nominal tip-to-tip path. Small static misalignments can be partly compensated through installation calibration, whereas larger or time-varying misalignments would require more rigid mounting or recalibration. In vibration environments, image blur, marker pose fluctuation, and radar pointing jitter may degrade repeatability and measurement stability. Illumination variations mainly influence the camera branch, although the use of marker-based localization and triggered acquisition improves robustness to moderate lighting changes. By contrast, the radar branch is largely insensitive to illumination but may still be affected by reduced signal-to-noise ratio or clutter under unfavorable conditions. These factors are not fully reproduced on the present simplified simulation platform. Therefore, the current experimental results should be interpreted as validation under representative laboratory dynamic intersection conditions, while further verification under more realistic aeroelastic, vibration, and environmental conditions remains future work.

## 6. Conclusions

Addressing the risk of blade interference and collision in coaxial twin-rotor helicopters during high-speed rotation, this study conducted systematic research on establishing blade collision warning parameters and developing in situ dynamic measurement methods. By analyzing the relative motion characteristics of coaxial twin-rotor blades, a blade collision warning parameter expression centered on the minimum blade tip distance is proposed. A multi-sensor collaborative measurement scheme integrating encoder phase measurement, tip-tracking camera positioning, and millimeter-wave radar distance measurement was established to dynamically capture tip distances during blade overlap.

Methodologically, this study established a blade intersection criterion based on rotor phase information. It combines tracking visual measurement to obtain spatial tip position data and utilizes FMCW millimeter-wave radar for direct distance measurement at the instant of blade intersection. By integrating multi-source information within a unified temporal and coordinate framework, a blade collision warning parameter measurement model for reconstructing the blade collision warning parameter suitable for coaxial twin-rotor motion environments was developed. To validate the feasibility and measurement performance of the proposed method, a dynamic rotor motion simulation test platform was constructed, and systematic experiments were conducted under varying tip distances and rotational speeds. Experimental results demonstrate that under the representative dynamic intersection conditions provided by the simulation platform, and for maximum rotational speeds of approximately 420 r/min and with tip distances ranging from 99 to 990 mm, the proposed measurement method stably outputs blade collision warning parameters. The measurement error is controlled within ±1.87 mm, with the repeatability error not exceeding 0.67 mm with a coefficient of variation below 0.2%, validating the measurement accuracy and stability under the tested laboratory conditions. Additional multi-speed experiments further showed that, over the tested rotational speed range, the measurement error of the blade collision warning parameter remains within (−5.86 mm, 6.57 mm), and the maximum absolute error does not exceed 6.57 mm. Although the fluctuation of the results increased moderately at higher speeds as the blade intersection duration becomes shorter, the proposed system maintained stable operation throughout the tested speed range.

It should be emphasized that the present validation was conducted on a simplified dynamic simulation platform with single side rotation and rigid blades. Therefore, the results verify the feasibility of phase triggered acquisition, multi-sensor information fusion, and warning parameter reconstruction under representative dynamic intersection conditions, rather than under the full aeromechanical, aeroelastic, and flight-load conditions of an actual coaxial helicopter.

In summary, the proposed blade collision warning parameters and their multi-sensor fusion measurement method provide a feasible laboratory validated technical route for dynamic blade tip distance measurement and collision risk parameter reconstruction in coaxial twin-rotor systems. The present work established the measurement principle, system framework, and experimental basis for subsequent validation under more realistic rotor operating conditions.

Future research may further expand and deepen the research in the following aspects. First, at the measurement method level, introducing millimeter-wave radars with higher bandwidth or multi-antenna configurations is expected to enhance tip distance measurement resolution and improve adaptability to complex scattering environments. Second, at the system level, tighter integration of the proposed measurement method with flight control systems could explore real-time interference suppression and active control strategies based on measurement results. Additionally, the proposed method should be further evaluated on a more realistic counter-rotating twin-rotor platform and under conditions that more closely resemble in-flight aerodynamic loads, vibration environments, and attitude variations. Such studies will be essential for advancing the method toward engineering application.

## Figures and Tables

**Table 1:** Comparison of key dynamic parameter coverage between the experimental platform and the actual coaxial system.

Key Dynamic Parameter	Values of the Real System	Scope of the Platform
Adjustable range of blade tip distance	100–990 mm	100–1200 mm
Blade overlap duration	1 ms	<1 ms
Rotor speed	≈400 r/min	300–1500 r/min
Time interval between adjacent intersections	≈18.75 ms	5–25 ms

**Table 2:** Single-trial experimental measurement results of blade collision warning parameters based on simulation platform.

$\mathrm{Reference}\;\mathrm{Value}\;d_{R}$ (mm)	Measured Value d (mm)	Error Δd (mm)
99.00	98.24	−0.76
198.00	196.53	−1.47
297.00	298.34	1.34
396.00	396.24	0.24
495.00	496.87	1.87
594.00	593.39	−0.61
693.00	692.75	−0.25
792.00	793.65	1.65
891.00	890.58	−0.42
990.00	991.24	1.24

**Table 3:** Constructing intermediate variables for blade collision warning parameters.

$\mathrm{Blade}\;\mathrm{Tip}\;\mathrm{Distance}\;\mathrm{Measurement}\;\mathrm{Value}\;h_{meas}$ (mm)	$\mathrm{Tip}\;\mathrm{Position}\;\mathrm{Measurement}\;\mathrm{Value}\;\left(x_{E2},z_{E2}\right)$ (mm)	Pre-Cone Angle β (°)
99.23	(538.17, 34.22)	8.10
198.52	(538.85, 34.22)	8.12
301.35	(538.73, 34.28)	8.10
400.23	(538.96, 34.21)	8.10
501.89	(538.23, 34.30)	8.11
599.38	(538.19, 34.29)	8.11
699.77	(538.86, 34.25)	8.12
801.69	(538.75, 34.28)	8.12
899.58	(538.38, 34.31)	8.11
1001.23	(538.24, 34.22)	8.10

**Table 4:** Repeatability results of blade collision warning parameters based on experimental platform.

$\mathrm{Reference}\;\mathrm{Value}\;d_{R}$ (mm)	Mean Value $\bar{d}$ (mm)	Std. Value $\bar{d}$ (mm)	CoV (%)
99.00	98.75	0.18	0.18
198.00	198.23	0.2	0.10
297.00	296.88	0.19	0.06
396.00	396.11	0.34	0.09
495.00	494.26	0.25	0.05
594.00	594.64	0.67	0.11
693.00	693.26	0.46	0.07
792.00	792.61	0.32	0.04
891.00	892.01	0.41	0.05
990.00	990.42	0.53	0.05

## Data Availability

Data will be made available on request.
